# Factors associated with referral to physiotherapists for adult patients consulting for musculoskeletal disorders in primary care; an ancillary study to ECOGEN

**DOI:** 10.1186/s12875-023-01970-5

**Published:** 2023-01-14

**Authors:** M. Peurois, M. Bertin, N. Fouquet, N. Adjeroud, Y. Roquelaure, A. Ramond-Roquin

**Affiliations:** 1 Univ Angers, Univ Rennes, Inserm, EHESP, Irset (Institut de recherche en santé, environnement et travail) - UMR_S 1085, SFR ICAT, F-49000 Angers, France; 2 Univ Angers, Département de médecine générale, F-49000 Angers, France; 3 Santé publique France, Saint‑Maurice, France; 4 Univ Angers, CHU Angers, Univ Rennes, Inserm, EHESP, Irset (Institut de recherche en santé, environnement et travail) - UMR_S 1085, SFR ICAT, F-49000 Angers, France; 5 Département de Médecine de Famille Et de Médecine d’urgence, Université de Sherbrooke, Québec, Canada

**Keywords:** Physiotherapy, Musculoskeletal disorder, Primary health care, Multilevel analysis

## Abstract

**Background:**

Musculoskeletal disorders (MSD) are multifactorial requiring multidisciplinary treatment including physiotherapy. General practitioners (GP) have a central role in managing MSDs and mostly solicit physiotherapists accounting for 76.1% of physiotherapy referrals in France. Patient, physician, and contextual factors, including healthcare accessibility, can influence physiotherapy referral rates.

**Objective:**

To identify patient, physician, and contextual factors associated with physiotherapy referral in adult patients with MSDs in general practice.

**Methods:**

This study is based on the 2011/2012 French cross-sectional ECOGEN study. Analyses included working-age patients consulting their GP for any MSD. Physiotherapy referral was assessed initially, then adjusted multilevel logistic model analysis of patient, physician, geographical area-related factors associated with these referrals was performed.

**Results:**

Among the 2305 patients included, 456 (19.8%) were referred to a physiotherapist. Following multilevel multivariate analyses, physiotherapist referral was more frequent for female patients (OR 1.28; 95% CI [1.03, 1.59]) with spinal (OR 1.47; 95% CI [1.18, 1.83]) and upper limb disorders (OR 1.66; 95% CI [1.20, 2.29]), and less frequent for patients ≥ 50 years (OR 0.69; 95% CI [0.52, 0.91]), living in deprived geographical areas (OR 0.60; 95% CI [0.40, 0.90]). GPs referred to a physiotherapist less frequently if they were ≥ 50 years (OR 0.50; 95% CI [0.39, 0.63]), had a high number of annual consultations, or were practicing in semi-urban area in a multidisciplinary team.

**Conclusion:**

This multilevel analysis identifies factors associated with physiotherapy referral for patients with MSDs, including living in deprived geographical areas. This constitutes an original contribution towards addressing healthcare disparities.

**Supplementary Information:**

The online version contains supplementary material available at 10.1186/s12875-023-01970-5.

## Background

Musculoskeletal disorders (MSDs) affect the musculoskeletal system (i.e. muscles, tendons, ligaments, nerves, discs, cartilage, and joints) and can be caused by several contributors such as repetitive strain during work or sport, accidents, ageing or congenital conditions. In France, MSDs account for 50% of occupational accident compensation and 87% of occupational disease compensation [1, 2]. Similarly, in European countries, nearly 50% of employees reported MSD symptoms within the past 12 months with the most common being low back pain (LBP) (44%) and upper-limb pain (42%) [3]. The economic burden is estimated at 3% of gross domestic product, with 10 million working days lost in France alone and 1 billion euros paid in compensation in 2012 [4, 5]. The human burden is also considerable, with LBP being the leading global cause of years lived with disability for both genders in 2017, followed by headache and depression [6, 7].

MSDs are multifactorial in origin involving biomechanical, professional, psychosocial, or work organisation factors, and possibly follow an exposomic model [8, 9]. For this reason, treatment is based on a multidisciplinary, holistic approach, including physical activity and active physiotherapy, medication, surgery, psychotherapy, alternative medicine and, social and administrative procedures including sick leave and worker’s compensation [10].

General practitioners (GP) have a central role in managing MSDs. In a 2010 French survey, 77% of patients who had experienced LBP in the previous 2 months had consulted a GP [11]. Faced with MSDs, GPs mostly solicit physiotherapists with GP referrals accounting for 76.1% of physiotherapy referrals in France [11, 12].

Physiotherapy has been shown to reduce pain, disability, opioid use, imaging investigations, medical or surgical consultations, infiltrations, and care-related costs [13–15]. For these reasons, European and American guidelines recommend early physiotherapy [16, 17].

However, observational studies showed that GPs have heterogeneous referrals habits to physiotherapists both within and between European countries, even for the same pathology, leading to a lack of coordination on MSD management, and potentially a poorer MSD management for patients [18]. Additionally, referring to a physiotherapist consultation can also be impacted by patient, physician and contextual factors including psychosocial mechanisms or healthcare system accessibility [19, 20].

This study aimed to identify patient, GP, and contextual factors associated with physiotherapy referral in France among 18 to 65-year-old patients consulting for MSDs in general practice to highlight geographical inequalities in French health accessibility and areas for improvement in territorial policies.

## Methods

### Study design

This is an ancillary study to ECOGEN (Elements of COnsultation in GENeral practice), which is a French national, cross-sectional, observational study conducted by the French College of General Practice Teachers (CNGE) between November 2011 and April 2012. The ECOGEN study design has been previously described [21]. It aimed to describe reasons for encounter, consultation results and healthcare procedures in primary care setting, over 20 613 consultations of general practice. Fifty-four trainee GPs collected data during their general practice internship with 128 GPs who were internship supervisors affiliated to 27 French medical schools. The trainees underwent a 2-day centralised data collection training course.

### Ethical considerations

The ECOGEN study was approved by an ethics committee (CPP Sud-Est L11-149, 10/11/2011) and included consent for ancillary studies on the ECOGEN database. A poster in the waiting room was used to inform patients about the study. GPs presented the study to their patients at the start of the consultation and written informed consent was obtained.

### ECOGEN Data collection

The ECOGEN study captured data from all consultations on one day per week. Specifically, patient age, gender, socio-professional category, receipt of compensation (occupational accident or disease), reason for consultation, consultation results (health conditions managed during the consultation) and prescribed healthcare procedures. The verbatim and data were collected on a paper form and coded using the ICPC-2 classification (International Classification of Primary Care, 2^nd^ edition, proposed by the WONCA) according to a hierarchical structure [22] enabling consultation results and healthcare procedures to be classified by body system. They were then entered into a centralised online database. Double data collection was performed on one day to ensure reproducibility and minimise error for each investigator.

A consultation could produce one or several consultation results, and each consultation result can lead to one or several healthcare procedures defined as a clinical examination, imaging, laboratory assessment, prescription for medication or sick-leave, referral to another physician or allied health professional, advice, or recommendations.

### Inclusion criteria

For our ancillary study, the analyses included working-aged patients aged from 18 to 65 years with a consultation result coded for one of the MSD codes. These MSD codes were selected from the Locomotor “L” category including conditions resulting from overuse of the musculoskeletal system (Additional file 1). Infectious, inflammatory, traumatic, and neoplastic codes were not included. Patients out of these ages were not included because of the working related variables we aimed to study.

We identified relevant clinical variables and potential confounders using a Directed Acyclic Graph (DAG) based on the literature findings (Additional file 2).

### Contextual variable aggregation

To approach patients’ contextual data on a pertinent geographical scale, the French national institute of statistics and economic studies (INSEE) proposed to study these geographical variables on a mesoenvironment level [23]. GP and physiotherapist accessibility was estimated using a geographical “catchment area” that included several towns where at least 16 out of the 31 social and health facilities were available [23]. These facilities included educational and health services, personal services such as hairdressers, retail/sports/culture/leisure facilities and transport infrastructure. Off-peak travel time was used to assess patient proximity to these social and health facilities, and geographical areas were then determined using an iterative aggregation method developed by the INSEE [23].

The two calculated contextual variables relating to the *catchment area* were GP and physiotherapist accessibility and the French deprivation index (FDep) based on the patient postcode. Data were available at town level [24], and were aggregated at geographical catchment level using a weighted mean.

a) *GP and physiotherapist accessibility.* Health system decision-makers use healthcare professional accessibility as an accessibility indicator, known as potential localised accessibility (PLA), which is calculated using an iterative aggregation method [23]. A PLA of 1 equates to a full-time GP practicing in a location 15 min away from the patient.

b) *French deprivation index.* The French Deprivation index, proposed by Rey et al. in 2009 [25], was used to assess social inequalities at a geographical level. This index is based on a Principal Component Analysis associating median incomes and the proportion of the population who are employed, unemployed and have a secondary education diploma. The score increases with the deprivation markers.

### Analyses

MSD consultations were identified and characteristics for patients with and without physiotherapy referral were compared, for age, gender, profession, compensation for an occupational accident or disease, mean consultation duration, time of day, and healthcare procedures (laboratory assessment, imaging, infiltration, medication, sick leave prescription, advice).

Quantitative independent patient variables were compared using Student’s t-test, or a non-parametric Mann–Whitney U test in case of variance inequality. Qualitative variables were described using frequencies and percentages and compared using the Chi-squared test or Fisher’s exact test for small numbers (*n* < 5).

Physiotherapy referral probability was modelled according to a marginal adjusted logistic model based on Generalised Estimating Equations (GEE) with an exchangeable variance–covariance matrix, due to the hierarchical data structure and the population-average approach [26, 27]. Marginal and mixed univariate analyses were performed for all the variables identified by the DAG, with a statistical α threshold of 0.20. An adjusted multilevel analysis was then performed with the univariate variables retained, with a statistical α threshold of 0.05. Interactions between patient age and spine or rotator cuff tendinitis symptoms, and between the FDep and these symptoms were also tested in the multivariate analyses.

Sensitivity analyses were performed last to compare our marginal model with both a random intercept model and a fixed slope mixed model on physician and geographical area variables. Two sub models including only spine or shoulder MSD were also performed, following the same method.

Statistical analyses were performed using R software, version 1.1.463, and the packages joineR, dplyr, stringr, car, FactoMineR, factoextra, lme4, survival, ICC, geepack, gee.

## Results

### Description

**Fig. 1:**
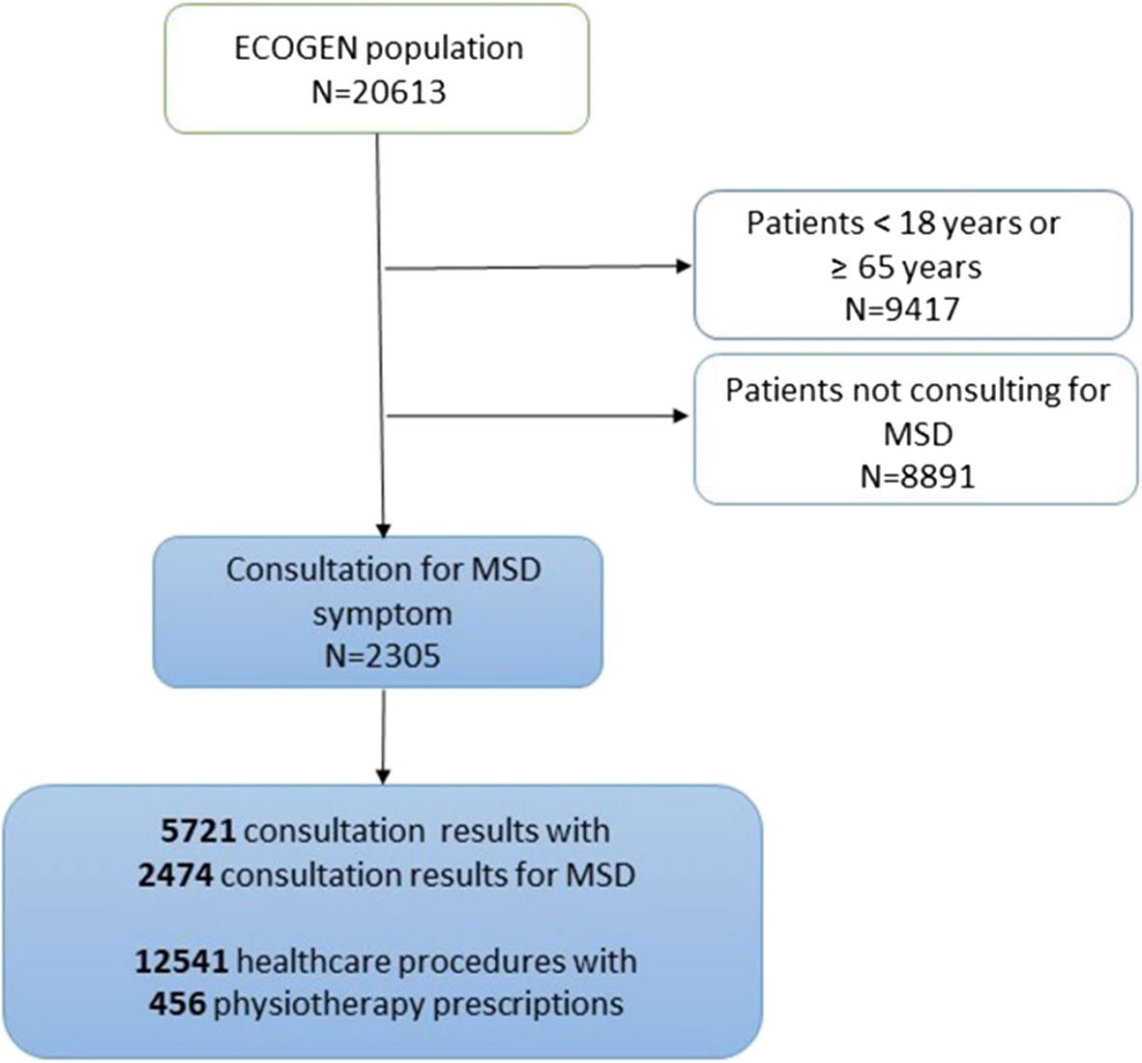
Flow chart showing inclusion of 2305 patients with musculoskeletal symptoms

Among the 11,196 patients aged from 18 to 65 years, 2305 (20.6%) consulted for an MSD symptom (Fig. 1).

The most frequent MSD symptoms for these 2305 patients were LBP (31.4%), shoulder pain (10.3%) and cervical pain (7.9%) (Additional file 3). Of these patients, 6.9% presented multi-site pain, 12.6% muscular pain and 11.0% arthrosis. Overall, spinal symptoms made up 44.9% of all MSD symptoms, upper limb 16.7% and lower limb 9.8%.

Among the 2305 MSD patients, 456 (19.8%) were referred to a physiotherapist. Patients referred for physiotherapy were more frequently women (*p* = 0.024) aged under 50 years (*p* = 0.03), compared with those who were not referred. There was no statistical difference between physiotherapy referral for MSD and profession or consultation duration (Table 1). The MSD sites with the highest physiotherapy referral rates were cervical (28.9%), shoulder (25.7%), back (23.7%), lumbar (22.4%) and elbow (22.5%).

**Table 1:** Characteristics of 2305 patients consulting for musculoskeletal disorders according to physiotherapy referral status

	**Total population with MSD symptoms**	**Physiotherapy referral**		**No physiotherapy referral**		***p*-value**
**Patient variables**	***N* = 2305 (%)**	***N* = 456 (%)**	**Confidence interval 95%**	***N* = 1849 (%)**	**Confidence interval 95%**	
**Age, n (%)**						**0.003**
< 35 years	439 (19.0)	110 (24.1)	[20.2 – 28.0]	329 (17.8)	[16.1 – 19.5]	
35–50 years	755 (32.8)	152 (33.3)	[29.0 – 37.7]	603 (32.6)	[30.5 – 34.7]	
> 50 years	1111 (48.2)	194 (42.6)	[38.0 – 47.1]	917 (49.6)	[47.3 – 51.9]	
**Gender:** Female, n (%)	1382 (60.0)	295 (64.7)	[60.3 – 69.1]	1087 (58.8)	[56.5 – 61.0]	**0.024**
**Profession, n(%)**						**0.049**
Farmer	17 (0.7)	2 (0.4)	[0.0 – 1.0]	15 (0.8)	[0.4 – 1.2]	
Self-employed	129 (5.6)	24 (5.3)	[3.2 – 7.3]	105 (5.7)	[4.6 – 6.7]	
Managerial staff	169 (7.3)	28 (6.1)	[3.9 – 8.3]	141 (7.6)	[6.4 – 8.8]	
Intermediate-level profession	201 (8.7)	56 (12.3)	[9.3 – 15.3]	145 (7.8)	[6.6 – 9.1]	
Salaried worker	898 (39.0)	183 (40.1)	[35.6 – 44.6]	715 (38.7)	[36.4 – 40.9]	
Manual worker	241 (10.5)	49 (10.7)	[7.9 – 13.6]	192 (10.4)	[8.0 – 11.8]	
Retired	302 (13.1)	46 (10.1)	[7.3 – 12.9]	256 (13.8)	[12.3 – 15.4]	
Unemployed	348 (15.1)	68 (14.9)	[11.6 – 18.2]	280 (15.1)	[13.5 – 16.8]	
**Compensation for an occupational accident or disease, n (%)**	247 (10.7)	50 (11.0)	[8.1 – 13.8]	197 (10.7)	[9.2 – 12.1]	0.914
**Consultation duration,** mean (standard deviation)	18.23 (11.69)	18.13 (7.94)	[17.4—18.9]	18.25 (12.45)	[17.7 – 18.8]	0.800

Interpretation: Percentages are distributed in column. For example: among the 2 305 patients with MSD symptoms, 24.1% of the patients who were referred to a physiotherapist were aged under 35 years, versus 17.8% of the patients who were not referred to a physiotherapist

*MSD* Musculoskeletal disorder

Medication (64.5%), imaging (17.1%) and sick leave (16.9%) were most frequently prescribed for MSD while laboratory investigations and infiltrations were scarce (Table 2). Physiotherapy referral frequencies decreased with the number of other associated healthcare procedures (p < 0.001).

**Table 2:** Healthcare procedures associated with consultation for musculoskeletal disorder symptoms in 2305 patients

	**Total population with MSD symptoms**	**Physiotherapy referral**		**No physiotherapy referral**		***p*-value**
	***N* = 2305 (%)**	***N* = 456 (%)**	**Confidence intervals 95%**	***N* = 1849 (%)**	**Confidence intervals 95%**	
**Number of healthcare procedures associated with an MSD symptom per patient, n(%)**						** < 0.001**
1–3	846 (36.7)	274 (60.1)	[ 55.6—64.6]	572 (30.9)	[ 28.8—33.0]	
4–6	976 (42.3)	128 (28.1)	[ 23.9—32.2]	848 (45.9)	[ 43.6—48.1]	
> 6	483 (21.0)	54 (11.8)	[ 8.9—14.8]	429 (23.2)	[ 21.3—25.1]	
**Healthcare procedures, n(%)**						
Medication	1486 (64.5)	262 (57.5)	[ 52.9—61.0]	1224 (66.2)	[ 64.0—68.4]	** < 0.001**
Imaging	394 (17.1)	56 (12.3)	[ 9.3—15.3]	338 (18.3)	[ 16.5—20.0]	**0.002**
Sick leave	390 (16.9)	73 (16.0)	[ 12.6—19.4]	317 (17.1)	[ 15.4—18.9]	0.562
Advice and recommendations	224 (9.7)	56 (12.3)	[ 9.3—15.3]	168 (9.1)	[ 7.8—10.4]	0.039
Laboratory investigation	62 (2.7)	3 (0.7)	[ 0.0—1.4]	59 (3.2)	[ 2.4—3.0]	**0.001**
Infiltration	51 (2.2)	3 (0.7)	[ 0.0—1.4]	48 (2.6)	[ 1.9—3.3]	**0.008**

*MSD* Musculoskeletal disorder

### Hierarchical model for patients consulting for an MSD

Table 3 presents the results for the GEE models. Patients were more likely to be referred to a physiotherapist if they were women and if they presented a spinal or upper limb symptom. However, physiotherapy referral was less likely if the patient was aged over 50 years, lived in an area with a high FDep, or had four or more associated healthcare procedures. GPs were less likely to refer to a physiotherapist if they were over 50 years old, practicing in a semi-urban area or in a multidisciplinary team or had a high annual number of consultations (over 5000/year).

**Table 3:** Associations determined using an adjusted logistical GEE model

**Variable**	**Model considering all MSD location (*n* = 2305)** OR (CI 95%)	***p*-value**	**Model considering only spinal location (*n* = 906)** OR (CI 95%)	***p*-value**	**Model considering only shoulder location (*n* = 255)** OR (CI 95%)	***p*-value**
**Patient variables**						
**Patient age**						
18–34 years	1		1		1	
35–50 years	0.78 (0.58–1.04)	0.084	0.78 (0.59–1.05)	0.096	0.75 (0.56–1.01)	0.056
50–65 years	0.69 (0.52–0.91)	**0.008**	0.74 (0.56–0.98)	**0.033**	0.65 (0.49–0.86)	**0.003**
**Gender** female	1.28 (1.03–1.59)	**0.024**	1.30 (1.05–1.62)	**0.011**	1.29 (1.04–1.60)	**0.020**
**Number of associated healthcare procedures**						
0–3	1		1		1	
4–6	0.73 (0.57–0.93)	**0.013**	0.73 (0.57–0.93)	**0.016**	0.73 (0.57–0.94)	**0.014**
> 6	0.68 (0.50–0.94)	**0.018**	0.70 (0.51–0.96)	**0.027**	0.69 (0.50–0.95)	**0.022**
**GP variables**						
**GP age** > 50 years	0.50 (0.39–0.63)	** < 0.001**	0.50 (0.40–0.63)	** < 0.001**	0.50 (0.39–0.63)	** < 0.001**
**Practice location**						
Rural	1		1		1	
Semi-urban	0.62 (0.42–0.90)	**0.013**	0.62 (0.43–0.91)	**0.018**	0.61 (0.42–0.90)	**0.011**
Urban	0.84 (0.58–1.21)	0.344	0.85 (0.59–1.23)	0.325	0.83 (0.57–1.20)	0.319
**Type of practice**						
Alone	1		1		1	
Group	0.98 (0.74–1.30)	0.902	0.98 (0.74–1.30)	0.806	0.99 (0.74–1.31)	0.924
Multidisciplinary team	0.62 (0.43–0.90)	**0.011**	0.62 (0.43–0.90)	**0.009**	0.63 (0.43–0.91)	**0.014**
**Number of consultations per year** > 5000	0.79 (0.63–0.99)	**0.038**	0.80 (0.64–1.00)	0.052	0.78 (0.62–0.98)	**0.032**
**Geographical variables**						
**FDep**						
Q1	1		1		1	
Q2	0.73 (0.52–1.00)	0.053	0.74 (0.53–1.02)	0.079	0.73 (0.52–1.01)	0.054
Q3	0.61 (0.41–0.90)	**0.013**	0.62 (0.42–0.92)	**0.011**	0.60 (0.40–0.89)	**0.011**
Q4	0.60 (0.40–0.90)	**0.013**	0.61 (0.40–0.91)	**0.009**	0.59 (0.39–0.89)	**0.012**
**Spine symptoms (versus any other)**	1.47 (1.18–1.83)	** < 0.001**	-	**-**	-	-
**Shoulder symptoms (versus any other)**	1.66 (1.20–2.29)	**0.002**	-	-	-	**-**

Associations between physiotherapy referral for musculoskeletal disorders, patient and GP characteristics and contextual characteristics according to an adjusted logistical GEE model

*MSD* musculoskeletal disorder, *OR* Odds ratio, *FDep* French Deprivation index, Q1: 1^st^ quartile, Q2: 2^nd^ quartile, Q3: 3^rd^ quartile, Q4: 4^th^ quartile (deprivation gradient from the least to the most deprived area)

Furthermore, multivariate analyses identified similar associations when analyses were restricted to the spinal diagnosis. However, there was a negative association between physiotherapist accessibility and physiotherapy referral (Additional file 4). In contrast, no associations were observed between the selected factors and shoulder symptoms (Additional file 5).

Sensitivity analyses with mixed effect models found similar results (Additional files 6 and 7).

## Discussion

### Main findings

In the present study, one in five (19.8%) patients consulting a French GP for MSD symptoms were referred to a physiotherapist. Physiotherapy referral was directly associated with a combination of factors related to the patient, the GP, and territorial characteristics. Specifically, younger, female patients were more likely to have a physiotherapy referral, whereas, physiotherapy referral was less likely with older physician age, semi-urban practice location, multidisciplinary practice, older patients, larger numbers of healthcare procedures and increased deprivation.

### Comparison with existing literature

Few other studies examining physiotherapy referral are available. The existing literature has already suggested these patient characteristics among patients with chronic LBP or MSD [28, 29] while few studies have explored the impact of GP and contextual characteristics. Long consultation duration, the existence of compensation for occupational disease or accident, and physicians practicing in a rural area are other factors shown to be associated with increased physiotherapy referral in the literature and in our study [30–32]. These studies also reported an association between physiotherapy referral and socio-economic factors including private health insurance, or pain management with co-prescription of non-steroidal anti-inflammatory drugs and muscle relaxants.

In our study, MSD management varies between consultations with and without physiotherapy referral. When physiotherapy was proposed, there were significantly fewer prescriptions for laboratory and imaging investigations and medication. The healthcare procedures associated with MSD seem to suggest there are two distinct approaches to managing MSD based on the underlying diagnosis: either a functional approach in which physiotherapy is proposed but no further diagnostic exploration is required, or a biomedical approach with laboratory or imaging investigations, more medication and corticosteroid infiltrations. This biomedical approach could be explained by the uncertainty of the MSD diagnosis, the presence of disease complications or a patient’s specific pathology [33, 34]. Notably, the number of healthcare procedures could be associated with MSD management, or with the patient’s comorbidities.

### Strengths and limitations

The ECOGEN study is a French national, multicentre, observational study which included 20,613 patients in a primary care setting. The response rate was very high (99.2%), and missing data and coding error rates were very low (1.5%). This is one of the first French primary care observational studies to explore the hierarchical context of consultations (reason for consultation, consultation results and healthcare procedures) using the ICPC-2, and to our knowledge is the widest one to date. We therefore believe that the ECOGEN data remain highly valuable despite their age. Previous analyses based on this study did not reveal sociodemographic differences between ECOGEN physicians and French physicians nationally [21]. However, GP internship supervisors have been described as having particular prescription characteristics, such as prescribing more preventive treatments [35]. It could also be hypothesised that they follow recommendations and guidelines more closely.

Our study explored original variables in the primary care and public health context, using geographical variables (physiotherapist and GP accessibility, neighbourhood deprivation index) in a multilevel marginal approach. Sensitivity analyses comparing marginal, random and fixed effect models enhanced the robustness of the results and the internal validity of our study.

The ICPC-2 is suboptimal for MSD-related diagnoses, as it is less precise than other classifications, such as ICD 10 or DSM 4, but it was developed for the primary care context where diagnosis is often uncertain and consultation duration short [22]. In addition, this study is only representative of the French system, which has its own specificities in terms of prescription, referrals, MSD management, and cultural and economic factors.

These ECOGEN results reflect the French primary care and health system setting, where 76% of physiotherapy referrals are from GPs. In France, as in other countries, physiotherapy referral can be initiated by physiotherapists themselves or through self-referral. Self-referral is associated with lower healthcare costs and reduces consultation pressure on GPs [36]. Young patients with spine or shoulder pain in a sports or leisure context are most likely to self-refer [37]. Physiotherapy triage by a nurse in primary care setting has also been associated with lower healthcare costs, less pain and disability, reduced risk of chronicity and improved quality of life [38].

Finally, due to the cross-sectional data collection design, conclusions on causality are limited compared to a cohort study.

### Perspectives for clinical practice and research

Accessibility has been defined by Penchansky within 5 dimensions: availability (offer and needs), accessibility (geographical), accommodation (appointments, buildings), acceptance (social) and affordability (economic) [39]. Our study suggests association between physiotherapy referral, GP’s characteristics and deprivation index. In order to improve accessibility to healthcare it would be interesting to take into account these elements for patients with MSD, by promoting interprofessional collaboration between GP and physiotherapists, and by paying attention to patients’ deprivation markers (e.g. health, housing, nutrition, work, education, social relationships) that could interfere with MSD genesis and management. Interventional studies in primary care studying the impact of interprofessional training and improvement of healthcare accessibility in precarious territories are currently underway in France.

## Conclusion

Our study highlights the association between GP and contextual factors on physiotherapy referral rates for patients consulting for MSD, including living in deprived geographical areas. These findings suggest French territorial healthcare disparities that should be considered in a health inequality reduction approach. 

## Supplementary Information

**Additional file 1.** ICPC-2 codes inclusion criteria.

**Additional file 2. **Directed acyclic graph.

**Additional file 3. **Physiotherapy referral according to symptom location.

**Additional file 4**. Hierarchical model for spine-related MSDs.

**Additional file 5. **Univariate analyses for patients with rotator cuff tendinitis-related MSDs.

**Additional file 6. **Mixed model with random effects on physician and geographical catchment area.

**Additional file 7. **Mixed model with fixed effects on physician and geographical area.

## Data Availability

Prof Laurent Letrilliart, the ECOGEN study principal investigator, gave permission for ECOGEN study data to be accessed and provided the required data directly. The datasets analysed during the current study are available from the corresponding author on reasonable request.

## Abbreviations

MSD: Musculoskeletal Disorders
LBP: Low Back Pain
GP: General Practitioner
ECOGEN: Elements of COnsultation in GENeral practice
CNGE: French College of General Practice Teachers
ICPC: International Classification of Primary Care
WONCA: World Organisation of Family Doctors
INSEE: French National Institute of Statistics and Economic Studies
FDep: French Deprivation Index
PLA: Potential Localised Accessibility
GEE: Generalised Estimating Equations
DAG: Direct Acyclic Graph
ICD: International Classification of Diseases
DSM: Diagnostic and Statistical Manual of Mental Disorders
