# Supplementary material for: Factors associated with referral to physiotherapists for adult patients consulting for musculoskeletal disorders in primary care; an ancillary study to ECOGEN
Source: BMC Prim Care. 2023 Jan 14;24:13. doi: 10.1186/s12875-023-01970-5 (PMC9840270; doi:10.1186/s12875-023-01970-5)
Supplement: Supplementary file 1 — Additional file 1. ICPC-2 codes inclusion criteria. [file 12875_2023_1970_MOESM1_ESM.docx]

**Additional file 1 –** ICPC-2 codes inclusion criteria

| **ICPC-2 Code label** | **Code** | **ICPC-2 Code label** | **Code** | **ICPC-2 Code label** | **Code** |
| --- | --- | --- | --- | --- | --- |
| Neck symptom | L01 | Leg symptom | L14 | Osteoarthrosis of hip | L89 |
| Back symptom | L02 | Knee symptom | L15 | Osteoarthrosis of knee | L90 |
| Low back symptom | L03 | Ankle symptom | L16 | Osteoarthrosis other | L91 |
| Shoulder symptom | L08 | Foot symptom | L17 | Shoulder syndrome | L92 |
| Arm symptom | L09 | Muscle symptom | L18 | Tennis elbow | L93 |
| Elbow symptom | L10 | Neck syndrome | L83 | MSD other | L99 |
| Wrist symptom | L11 | Low back syndrome without radiating pain | L84 | Carpal tunnel syndrome | N93 |
| Hand/finger symptom | L12 | Low back syndrome with radiating pain | L86 | Peripheral neuropathy | N94 |
| Hip symptom | L13 | Bursitis/tendinitis/synovitis | L87 |  |  |
