# Supplementary material for: Factors associated with referral to physiotherapists for adult patients consulting for musculoskeletal disorders in primary care; an ancillary study to ECOGEN
Source: BMC Prim Care. 2023 Jan 14;24:13. doi: 10.1186/s12875-023-01970-5 (PMC9840270; doi:10.1186/s12875-023-01970-5)
Supplement: Supplementary file 2 — Additional file 2. Directed acyclic graph. [file 12875_2023_1970_MOESM2_ESM.docx]

**Additional file 2**: Directed acyclic graph


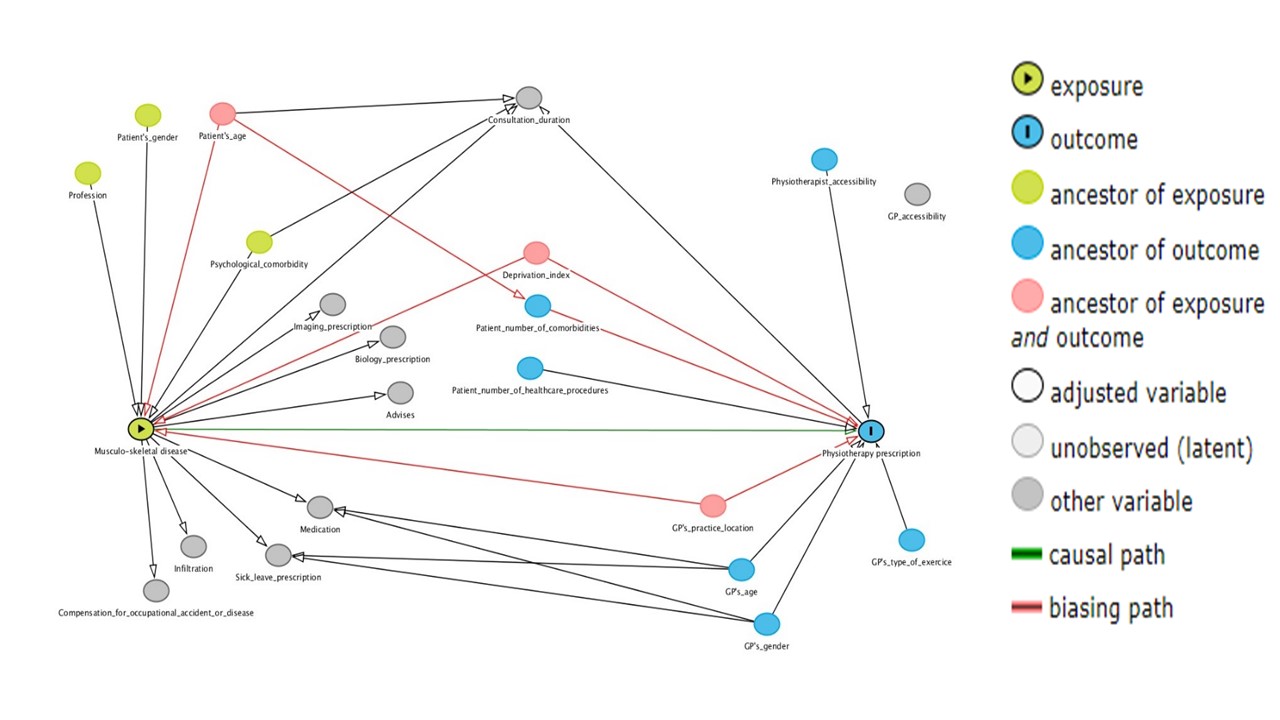


This directed acyclic graph is based on literature findings and was used to identify relevant clinical variables and potential confounders
