# Supplementary material for: Factors associated with referral to physiotherapists for adult patients consulting for musculoskeletal disorders in primary care; an ancillary study to ECOGEN
Source: BMC Prim Care. 2023 Jan 14;24:13. doi: 10.1186/s12875-023-01970-5 (PMC9840270; doi:10.1186/s12875-023-01970-5)
Supplement: Supplementary file 3 — Additional file 3. Physiotherapy referral according to symptom location. [file 12875_2023_1970_MOESM3_ESM.docx]

**Additional file 3 –** Physiotherapy referral according to symptom location

|  | **Total population with MSD symptoms (%)**  **N=2305** | **Physiotherapy referral (%)**  **N=456** | **No physiotherapy referral (%)**  **N=1849** | **p-value** |
| --- | --- | --- | --- | --- |
|  |  |  |  | **<0.001** |
| Cervical pain | 180 (7.9) | 52 (11.4) | 128 (6.9) |  |
| Back pain | 80 (3.5) | 19 (4.2) | 61 (3.3) |  |
| Low back pain | 723 (31.4) | 162 (35.5) | 561 (30.3) |  |
| Shoulder | 237 (10.3) | 61 (13.4) | 176 (9.5) |  |
| Elbow | 80 (3.5) | 18 (3.9) | 62 (3.4) |  |
| Wrist/Hand | 37 (1.6) | 4 (0.9) | 33 (1.8) |  |
| Hip | 33 (1.4) | 5 (1.1) | 28 (1.5) |  |
| Knee | 92 (4.2) | 8 (1.8) | 84 (4.5) |  |
| Leg/Foot | 83 (4.0) | 12 (2.6) | 71 (3.8) |  |
| Tendinitis/Myalgia | 290 (12.6) | 47 (10.3) | 243 (13.1) |  |
| Carpal tunnel syndrome | 48 (2.1) | 1 (0.2) | 47 (2.5) |  |
| Arthrosis | 254 (11.0) | 36 (7.9) | 218 (11.8) |  |
| Other | 8 (0.3) | 0 | 8 (0.4) |  |
| Multisite | 160 (6.9) | 31 (6.8) | 129 (7.0) |  |
