# Supplementary material for: Factors associated with referral to physiotherapists for adult patients consulting for musculoskeletal disorders in primary care; an ancillary study to ECOGEN
Source: BMC Prim Care. 2023 Jan 14;24:13. doi: 10.1186/s12875-023-01970-5 (PMC9840270; doi:10.1186/s12875-023-01970-5)
Supplement: Supplementary file 4 — Additional file 4. Hierarchical model for spine-related MSDs. [file 12875_2023_1970_MOESM4_ESM.docx]

**Additional file 4 –** Hierarchical model for spine-related MSDs

| **Variable** | **OR (CI 95%)** | **p-value** |
| --- | --- | --- |
| **Patient variables** |  |  |
| **Patient’s age**  35-50 years  >50 years | 0.77 (0.53-1.11)  0.66 (0.45-0.97) | 0.164  **0.034** |
| **Patient gender:** female | 1.49 (1.09-2.04) | **0.011** |
| **GP’s variables** |  |  |
| **Physician’s age** > 50 years | 0.47 (0.33-0.65) | **<0.001** |
| **Practice location**  Semi-urban  Urban | 0.40 (0.24-0.67)  0.61 (0.39-0.95) | **<0.001**  **0.027** |
| **Number of consultations**  >5000 per year | 0.67 (0.48-0.93) | **0.016** |
| **Geographical variables** |  |  |
| **Physiotherapist accessibility**  Q1  Q2  Q3 | 0.51 (0.30-0.85)  0.95 (0.65-1.39)  1.85 (1.95-3.5) | **0.010**  0.794  0.070 |

MSD: musculoskeletal disorder; OR: odds ratio; Q: quartile; bold = significant p-value
