# Supplementary material for: Factors associated with referral to physiotherapists for adult patients consulting for musculoskeletal disorders in primary care; an ancillary study to ECOGEN
Source: BMC Prim Care. 2023 Jan 14;24:13. doi: 10.1186/s12875-023-01970-5 (PMC9840270; doi:10.1186/s12875-023-01970-5)
Supplement: Supplementary file 5 — Additional file 5. Univariate analyses for patients with rotator cuff tendinitis-related MSDs. [file 12875_2023_1970_MOESM5_ESM.docx]

**Additional file 5 -** Univariate analyses for patients with rotator cuff tendinitis-related MSDs

| **Variable** | **Effectives**  **N=255** | **Physiotherapy referral (%)**  **N=67 (26.3)** | **OR (CI 95%)** | **p-value** |
| --- | --- | --- | --- | --- |
| **Patient variables** |  |  |  |  |
| **Patient’s age**  <35 years  35-50 years  50-65 years | 21  75  159 | 6 (28.6)  22 (29.3)  39 (24.5) | 1.00  1.04 (0.36-3.02)  0.81 (0.30-2.24) | -  0.946  0.688 |
| **Gender:** female | 150 | 38 (25.3) | 0.89 (0.51-1.56) | 0.681 |
| **Profession**  Farmer  Self-employed  Manager  Intermediate profession  Salaried worker  Manual Worker  Retired  Unemployed | 1  17  9  26  94  40  40  28 | 0  5 (29.4)  3 (33.3)  5 (19.2)  22 (23.4)  16 (40.0)  8 (20.0)  8 (28.6) | -  1.36 (0.43-4.36)  1.64 (0.39-6.82)  0.78 (0.26-2.31)  1.00  2.18 (0.99-4.80)  0.82 (0.33-2.02)  1.31 (0.51-3.36) | 0.988  0.596  0.510  0.653  -  **0.054**  0.666  0.578 |
| **Number of associated consultation results**  0  1-2  ≥3 | 82  137  36 | 24 (29.3)  33 (24.1)  10 (27.8) | 1.00  0.77 (0.41-1.43)  0.93 (0.38-2.26) | -  0.40  0.87 |
| **Compensation** for an occupational accident or disease | 48 | 17 (35.4) | 1.72 (0.89-3.32) | **0.1** |
| **Number of healthcare procedures**  1-3  4-6  >6 | 87  111  57 | 27 (31.0)  27 (24.3)  13 (22.8) | 1.00  0.71 (0.38-1.33)  0.66 (0.30-1.44) | -  0.288  0.292 |
| **Prescriptions**  Biology  Imagery  Medication  Infiltration  Advice  Sick leave | 152  2  55  19  19  47 | 33 (21.7)  0  10 (18.2)  8 (42.1)  1 (5.3)  14 (29.8) | 0.56 (0.32-0.99)  0.56 (0.26-1.19)  2.18 (0.83-5.71)  0.14 (0.02-1.11)  1.24 (0.62-2.50) | **0.045**  *  **0.13**  **0.11**  **0.061**  0.55 |
| **Consultation** duration >18 min | 110 | 30 (27.3) | 1.10 (0.62-1.92) | 0.75 |
| **Time of day**  Afternoon  Half-day end | 131  32 | 35 (26.7)  7 (21.9) | 1.05 (0.60-1.83)  0.76 (0.31-1.90) | 0.87  0.56 |
| **GP’s variables** |  |  |  |  |
| **GP’s age** > 50 years | 186 | 47 (25.3) | 0.83 (0.44-1.57) | 0.566 |
| **GP gender:** Female | 88 | 30 (34.1) | 1.82 (1.02-3.24) | **0.043** |
| **Practice location**  Rural  Semi-rural  Urban | 57  69  129 | 15 (26.3)  14 (20.3)  38 (29.5) | 1.00  0.71 (0.31-1.64)  1.17 (0.58-2.37) | -  0.426  0.664 |
| **Type of practice**  Alone  Group  Multidisciplinary team | 53  161  41 | 14 (26.4)  43 (26.7)  10 (24.4) | 1.00  1.02 (0.49-2.12)  0.90 (0.34-2.39) | -  0.967  0.823 |
| **Number of consultations by year** >5000 | 137 | 41 (29.9) | 1.51 (0.85-2.68) | **0.16** |
| **Geographical variables** |  |  |  |  |
| **Physiotherapist accessibility**  Q1  Q2  Q3  Q4 | 84  92  12  67 | 21 (25.0)  24 (26.1)  2 (16.7)  20 (29.9) | 0.78 (0.38-1.62)  0.83 (0.40-1.70)  0.47 (0.11-2.07)  1.00 | 0.509  0.610  0.319  - |
| **GP accessibility**  Q1  Q2  Q3  Q4 | 72  18  100  65 | 16 (22.2)  8 (44.4)  26 (26.0)  17 (26.2) | 0.81 (0.37-1.78)  2.26 (0.77-6.60)  0.99 (0.49-2.03)  1.00 | 0.595  **0.136**  0.983  - |
| **French Deprivation Index**  Q1  Q2  Q3  Q4 | 56  59  74  66 | 21 (37.5)  15 (25.4)  15 (20.3)  16 (24.2) | 1.00  0.57 (0.25-1.28)  0.42 (0.19-0.95)  0.53 (0.24-1.19) | **-**  **0.171**  **0.036**  **0.123** |

MSD: musculoskeletal disorder; OR: odds ratio; Q: quartile; bold=significant p-value
