# Supplementary material for: Factors associated with referral to physiotherapists for adult patients consulting for musculoskeletal disorders in primary care; an ancillary study to ECOGEN
Source: BMC Prim Care. 2023 Jan 14;24:13. doi: 10.1186/s12875-023-01970-5 (PMC9840270; doi:10.1186/s12875-023-01970-5)
Supplement: Supplementary file 6 — Additional file 6. Mixed model with random effects on physician and geographical catchment area. [file 12875_2023_1970_MOESM6_ESM.docx]

**Additional file 6 -** Mixed model with random effects on physician and geographical catchment area

|  | **Model considering all MSD location (n=2305)**  OR (CI 95%) | **p-value** | **Model considering only spinal location**  **(n=906)**  OR (CI 95%) | **p-value** | **Model considering only shoulder location (n=255)**  OR (CI 95%) | **p-value** |
| --- | --- | --- | --- | --- | --- | --- |
| Patient variables |  |  |  |  |  |  |
| **Patient’s age**  35-50 years  >50 years | 0.76 (0.57-1.03)  0.68 (0.51-0.91) | 0.078  **0.009** | 0.78 (0.57-1.05)  0.74 (0.55-0.99) | 0.097  **0.043** | 0.74 (0.55-1.00)  0.64 (0.48-0.86) | 0.050  **0.003** |
| **Patient gender**  Female | 1.26 (1.00-1.59) | **0.047** | 1.28 (1.01-1.61) | **0.037** | 1.27 (1.1-1.60) | **0.042** |
| **Consultation duration**  >18 min | 1.29 (1.02-1.64) | **0.034** | 1.30 (1.02-1.65) | **0.032** | 1.29 (1.02-1.64) | **0.036** |
| **Number of healthcare procedures**  4-6  >6 | 0.68 (0.53-0.88)  0.57 (0.41-0.79) | **0.004**  **<0.001** | 0.68 (0.52-0.88)  0.57 (0.41-0.79) | **0.003**  **<0.001** | 0.68 (0.53-0.88)  0.57 (0.41-0.79) | **0.004**  **<0.001** |
| **GP variables** |  |  |  |  |  |  |
| **Physician’s age**  >50 years | 0.54 (0.39-0.77) | **<0.001** | 0.55 (0.39-0.78) | **<0.001** | 0.55 (0.38-0.77) | **<0.001** |
| **Type of practice**  Group  Multidisciplinary team | 0.96 (0.64-1.44)  0.55 (0.32-0.94) | 0.847  **0.030** | 0.95 (0.64-1.43)  0.54 (0.31-0.93) | 0.821  **0.027** | 0.96 (0.64-1.44)  0.56 (0.33-0.97) | 0.857  **0.038** |
| **Geographical variables** |  |  |  |  |  |  |
| **FDep**  Q2  Q3  Q4 | 0.64 (0.33-1.22)  0.51 (0.28-0.92)  0.48 (0.27-0.84) | 0.173  **0.025**  **0.011** | 0.43 (0.68-1.07)  0.53 (0.33-0.85)  0.49 (0.31-0.78) | 0.092  **0.008**  **0.003** | 0.62 (0.33-1.17)  0.49 (0.27-0.88)  0.47 (0.26-0.83) | 0.139  **0.018**  **0.010** |
| **Spine symptoms (versus any other)** | 1.46 (1.16-1.82) | **0.001** |  |  |  |  |
| **Shoulder symptoms (versus any other)** | 1.77 (1.28-2.45) | **<0.001** |  |  |  |  |

FDep: French Deprivation Index; OR: odds ratio; Q: quartile; bold=significant p-value
