# Supplementary material for: Factors associated with referral to physiotherapists for adult patients consulting for musculoskeletal disorders in primary care; an ancillary study to ECOGEN
Source: BMC Prim Care. 2023 Jan 14;24:13. doi: 10.1186/s12875-023-01970-5 (PMC9840270; doi:10.1186/s12875-023-01970-5)
Supplement: Supplementary file 7 — Additional file 7. Mixed model with fixed effects on physician and geographical area. [file 12875_2023_1970_MOESM7_ESM.docx]

**Additional file 7 -** Mixed model with fixed effects on physician and geographical area

|  | **Model considering all MSD location (n=2305)**  OR (CI 95%) | **p-value** | **Model considering only spinal location**  OR (CI 95%) | **p-value** | **Model considering only shoulder location**  OR (CI 95%) | **p-value** |
| --- | --- | --- | --- | --- | --- | --- |
| Patient variables |  |  |  |  |  |  |
| **Patient’s age**  35-50 years  >50 years | 0.83 (0.62-1.12)  0.72 (0.54-0.96) | 0.231  **0.024** | 0.84 (0.63-1.14)  0.77 (0.57-1.03) | 0.270  **0.078** | 0.81 (0.60-1.09)  0.67 (0.50-0.90) | 0.16  **0.008** |
| **Patient gender**  Female | 1.30 (1.14-1.64) | **0.022** | 1.33 (1.06-1.67) | **0.015** | 1.31 (1.04-1.64) | **0.021** |
| **Number of healthcare procedures**  4-6  >6 | 0.73 (0.56-0.94)  0.63 (0.46-0.86) | **0.013**  **0.004** | 0.73 (0.56-0.94)  0.63 (0.46-0.87) | **0.013**  **0.005** | 0.73 (0.57-0.95)  0.64 (0.47-0.88) | **0.017**  **0.007** |
| **GP variables** |  |  |  |  |  |  |
| **Physician’s age**  >50 years | 0.65 (0.46-0.90) | **0.009** | 0.65 (0.47-0.91) | **0.011** | 0.65 (0.46-0.90) | **0.010** |
| **Type of practice**  Group  Multidisciplinary team | 0.88 (0.61-1.28)  0.60 (0.38-0.96) | 0.497  **0.034** | 0.87 (0.60-1.27)  0.59 (0.37-0.95) | 0.476  **0.028** | 0.89 (0.62-1.30)  0.62 (0.39-0.99) | 0.555  **0.047** |
| **Spine symptoms (versus any other)** | 1.41 (1.13-1.76) | **0.003** |  |  |  |  |
| **Shoulder symptoms (versus any other)** | 1.71 (1.24-2.36) | **0.001** |  |  |  |  |

OR: odds ratio; bold=significant p-value
